# Quantitative assessment of choriocapillaris flow deficits and type 1 macular neovascularization growth in age-related macular degeneration

**DOI:** 10.1038/s41598-023-35080-0

**Published:** 2023-05-26

**Authors:** Diogo Cabral, Ana C. Fradinho, Yi Zhang, Hao Zhou, Prithvi Ramtohul, Meera S. Ramakrishnan, Telmo Pereira, Ruikang K. Wang, K. Bailey Freund

**Affiliations:** 1 Vitreous Retina Macula Consultants of New York, 950 Third Ave, New York, NY 10022 USA; 2 iNOVA4Health, NOVA Medical School I Faculdade de Ciências Médicas, Universidade NOVA de Lisboa, Lisbon, Portugal; 3 Department of Ophthalmology, NYU Grossman School of Medicine, New York, NY USA; 4 Department of Bioengineering, University of Washington, Seattle, WA USA; 5 Department of Ophthalmology, University of Washington, Seattle, WA USA

**Keywords:** Retina, Data processing

## Abstract

During the past 15 years, new treatment paradigms for neovascular age-related macular degeneration (nvAMD) have evolved due to the advent of intravitreal anti-vascular endothelial growth factor (VEGF) therapy and rapid advances in retinal imaging. Recent publications describe eyes with type 1 macular neovascularization (MNV) as showing more resistance to macular atrophy than eyes with other lesion types. We sought to explore whether the perfusion status of the native choriocapillaris (CC) surrounding type 1 MNV influences its pattern of growth. To evaluate this effect, we analyzed a case series of 22 eyes from 19 nvAMD patients with type 1 MNV exhibiting growth on swept-source optical coherence tomography angiography (SS-OCTA) over a minimum follow-up of 12 months. We observed an overall weak correlation between type 1 MNV growth and CC flow deficits (FDs) average size (τ = 0.17, 95% CI [− 0.20, 0.62]) and a moderate correlation with CC FD % (τ = 0.21, 95% CI [− 0.16, 0.68]). Type 1 MNV was located beneath the fovea in most of the eyes (86%) and median visual acuity was 20/35 Snellen equivalent. Our results support that type 1 MNV recapitulates areas of CC blood flow impairment while serving to preserve foveal function.

## Introduction

Following the introduction of optical coherence tomography (OCT), the anatomic location of neovessels in eyes with neovascular age-relate macular degeneration (nvAMD) has become more relevant for classifying distinct neovascular subtypes^1,2^. Type 1 macular neovascularization (MNV) refers to neovessels originating from the choriocapillaris to enter the sub-retinal pigment epithelium (RPE) space^3^. As hypothesized by Grossniklaus and Green in 2004, type 1 MNV appears to provide nutritional support to the overlying RPE and photoreceptors^2,3^. A recent clinicopathologic correlation of type 1 MNV showed the sub-RPE neovessels to have a fenestrated endothelium and a vascular density comparable to that of the native choriocapillaris in regions beyond its margins^4^. Eyes with type 1 MNV present with better visual acuity than those with those with Type 2, 3 or mixed lesions. These eyes have greater resistance to macular atrophy and retain better long-term visual function^4–9^.

Advances in OCT-angiography (OCTA) have enabled in *vivo* evaluation of the choriocapillaris (CC) and type 1 MNV at a high resolution^10–12^. Blood flow in the CC has a mathematically defined structure that is amenable to analysis using CC flow deficits (FD) features^13^. Impairment of CC flow is associated with an increase in FD average size and has been observed to occur in the setting of nvAMD and complete RPE and outer retinal atrophy (cRORA)^13–19^. Long-term follow-up studies have shown that type 1 MNV lesions show varying growth rates, irrespective of the number of anti-VEGF treatments and exudative features^20,21^. Although some studies suggest that impairment of CC might have a key role in the development of MNV, evidence that type 1 MNV grows over areas with impaired CC flow, i.e. increased flow deficits size, is still lacking^17,19^. The present work aimed to evaluate the association between FD quantitative measurements and growth patterns of type 1 MNV using swept-source OCTA (SS-OCTA).

## Results

A total of 34 eyes from 31 patients met study inclusion criteria. Following an initial assessment of SS-OCTA scan quality, 4 eyes (12%) were excluded due to imaging artifacts. Subsequently, 8 eyes (24%) were excluded due to poorly defined margins of the type 1 MNV flow signal which precluding precise grading. The remaining 22 eyes from 19 patients (13 female patients) were evaluated. The mean patient age at the baseline visit was 76 ± 2 years (range 61–89 years). Sixteen patients (84%) had systemic hypertension controlled with medical treatment. Median BCVA was 0.2 (0.2–0.38) LogMAR (Snellen equivalent of 20/32) at baseline and 0.25 (0.2–0.4) LogMAR (Snellen equivalent of 20/35) at the last follow-up visit. At the baseline (initial SS-OCTA) visit, non-exudative type 1 MNV lesions were present in 4 eyes (18%), and treatment-naive exudative type 1 MNV was identified in 5 eyes (23%). In the remaining eyes, the mean interval since the first identification of type 1 MNV was 23 ± 5 months and the median interval from the previous visit was 5 ± 3 (4–7) weeks. The mean follow-up using SS-OCTA was 28 ± 3 months (range 10–55). The median number of yearly visits per eye was 1 ± 1 (1–3), i.e., 7 out of 22 eyes had more than one visit. All patients with non-exudative type 1 MNV at baseline converted to exudative nvAMD over their follow-up and received at least one anti-VEGF treatment. The median treatment interval between anti-VEGF treatment and the last visit was 5 ± 3 (4–7) weeks.

At baseline, the median and respective IQR of type 1 MNV surface area was 0.50 ± 1.20 mm^2^ (Q1 = 0.40 mm^2^ and Q3 = 1.60 mm^2^) and 1.61 ± 2.33 mm^2^ (Q1 = 0.78 mm^2^ and Q3 = 3.11 mm^2^) at the last follow-up. At baseline, type 1 MNV was localized under the central fovea in 14 eyes (64%). In the remaining 8 eyes, the neovascular network enlarged in the direction of the foveal center over follow-up in 4 cases (50%). We did not observe the development of cRORA above type 1 MNV in any study eyes over follow-up.

**Figure 1:**
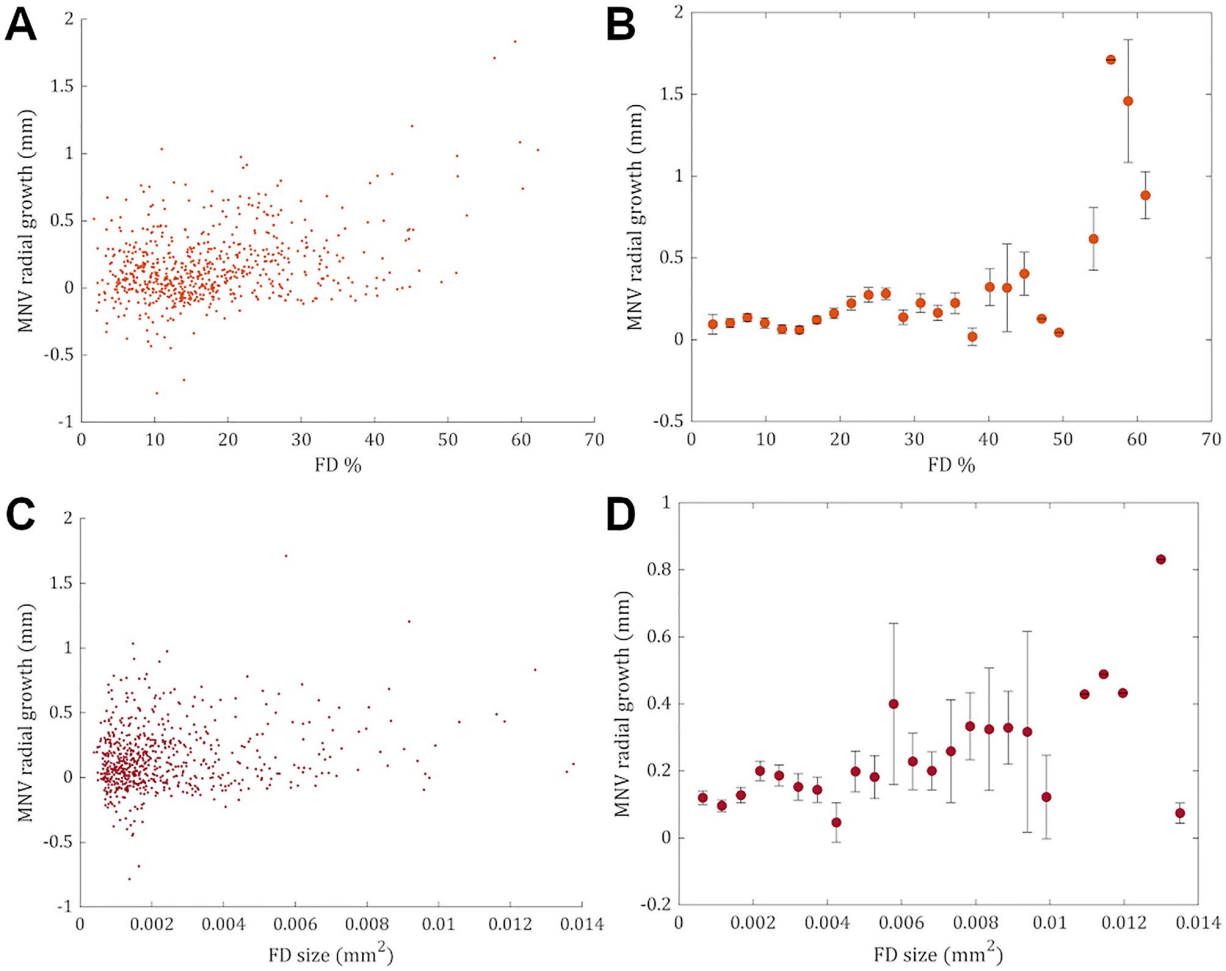
Data plots resulting from the analysis of the entire cohort. (**A**, **C**) Data plots resulting from the analysis of the entire cohort show a positive monotonic tendency between FD features (average size and percentage) and neovascular growth patterns, i.e., neovascular growth and the value of FD features moved in the same direction but not necessarily at a constant rate. (**B**, **D**) A monotonic relationship is better appreciated after binning FD features into uniform classes and plotting against the average neovascular growth (mean ± standard error of the mean) for each interval.

A visual depiction of the type 1 MNV growth patterns plotted against the CC FD features (% and average size) in the entire cohort is shown in Fig. 1. Inspection of data plots showed a positive monotonic relationship between FD features (average size and density) and neovascular growth, i.e., neovascular growth and the value of FD features moved in the same direction but not necessarily at a constant rate.

**Figure 2:**
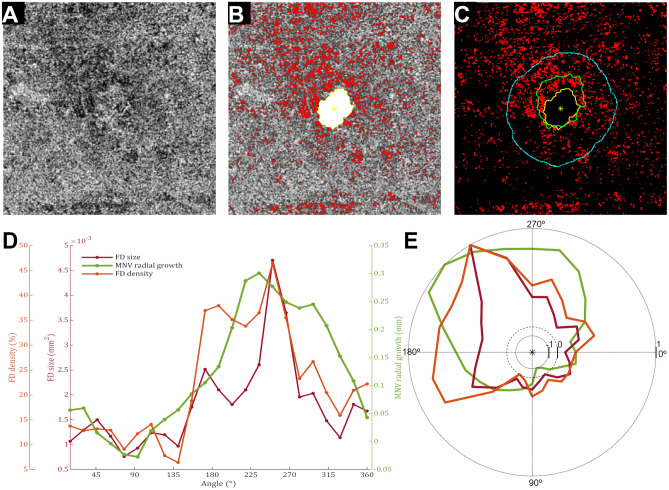
Single case analysis shows a positive correlation between choriocapillaris (CC) flow deficits (FDs) features and neovascular growth patterns. (**A**) *En face* choriocapillaris (CC) image was obtained using swept-source optical coherence tomography angiography. (**B**) False-colored CC flow deficits (FDs, red) were overlapped with macular neovascularization (MNV) segmentation (white), and its respective centroid (yellow asterisk) in the first visit. (**C**) Overlapping between CC FDs and MNV segmentation over follow-up (first visit, yellow; second visit, green) shows sectorial growth. (**D**) Visual inspection of data plots suggested a strong association between neovascular growth patterns and FD features, which was corroborated by statistical analysis (MNV growth and FD average size, τ = 0.61, p = 0.00001; MNV growth and FD percentage, τ = 0.59, p = 0.00003).

**Figure 3:**
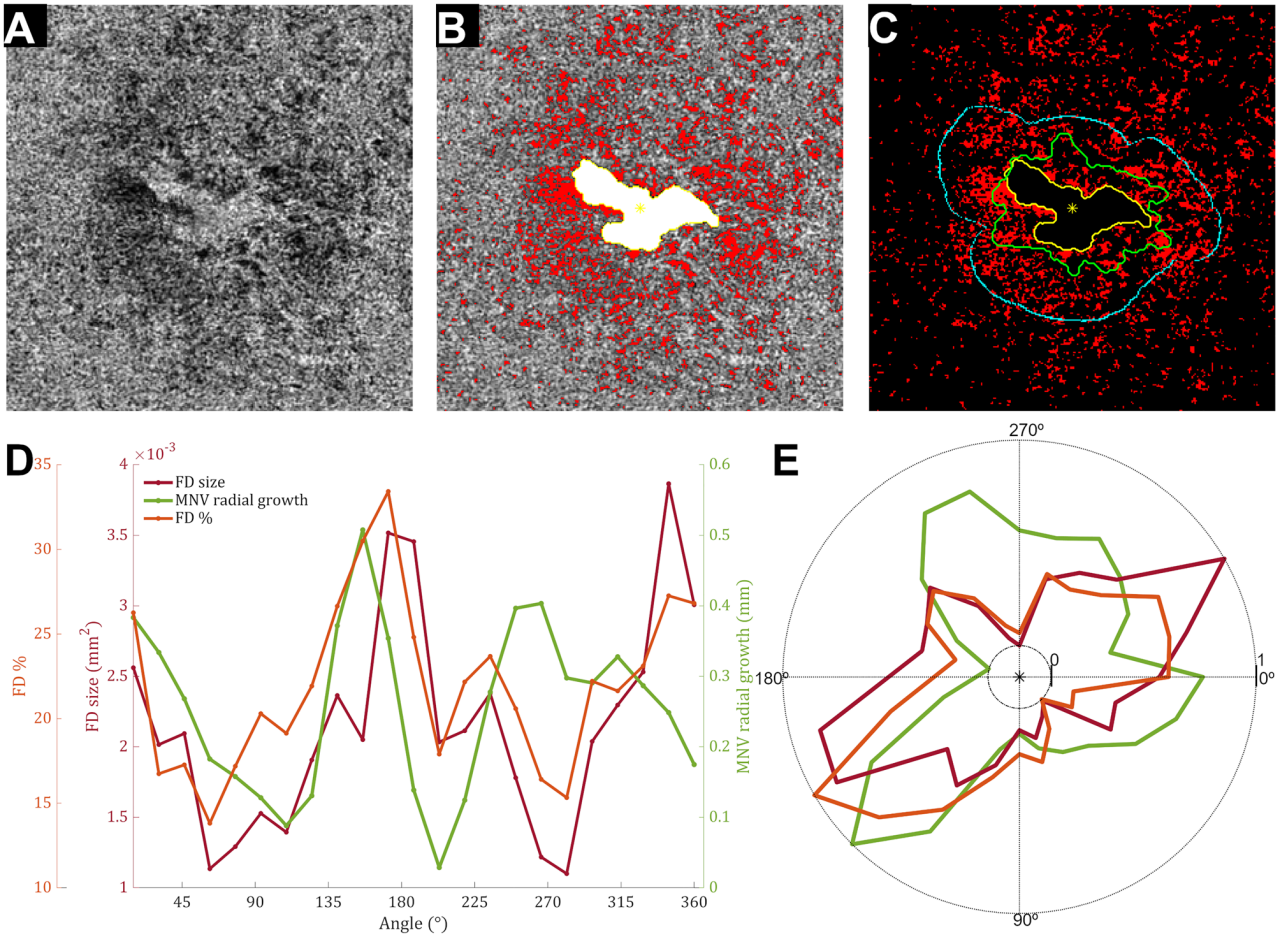
Single case analysis shows monotonic association between choriocapillaris (CC) flow deficits (FDs) features and neovascular growth patterns in half of the region of interest. (**A**) *En face* choriocapillaris (CC) image was obtained using swept-source optical coherence tomography angiography. (**B**) False-colored CC flow deficits (FDs, red) were overlapped with macular neovascularization (MNV) segmentation (white), and its respective centroid (yellow asterisk) at the first visit. (**C**) Overlapping CC FDs (red) with MVN segmentation over follow-up (first visit, yellow; second visit, green) shows growth within a range of 600 μm (cyan) from MNV limits at the first visit. (**D**) The plot of FD features (FD average size and FD percentage) and MNV patterns show a monotonic tendency between neovascular growth patterns and FD features within part of the image (0º–180º). Statistical analysis disclosed a very weak correlation between MNV growth and FD average size (τ = 0.04, p = 0.52) and a weak correlation between MNV growth and FD % (τ = 0.13, p = 0.40).

**Figure 4:**
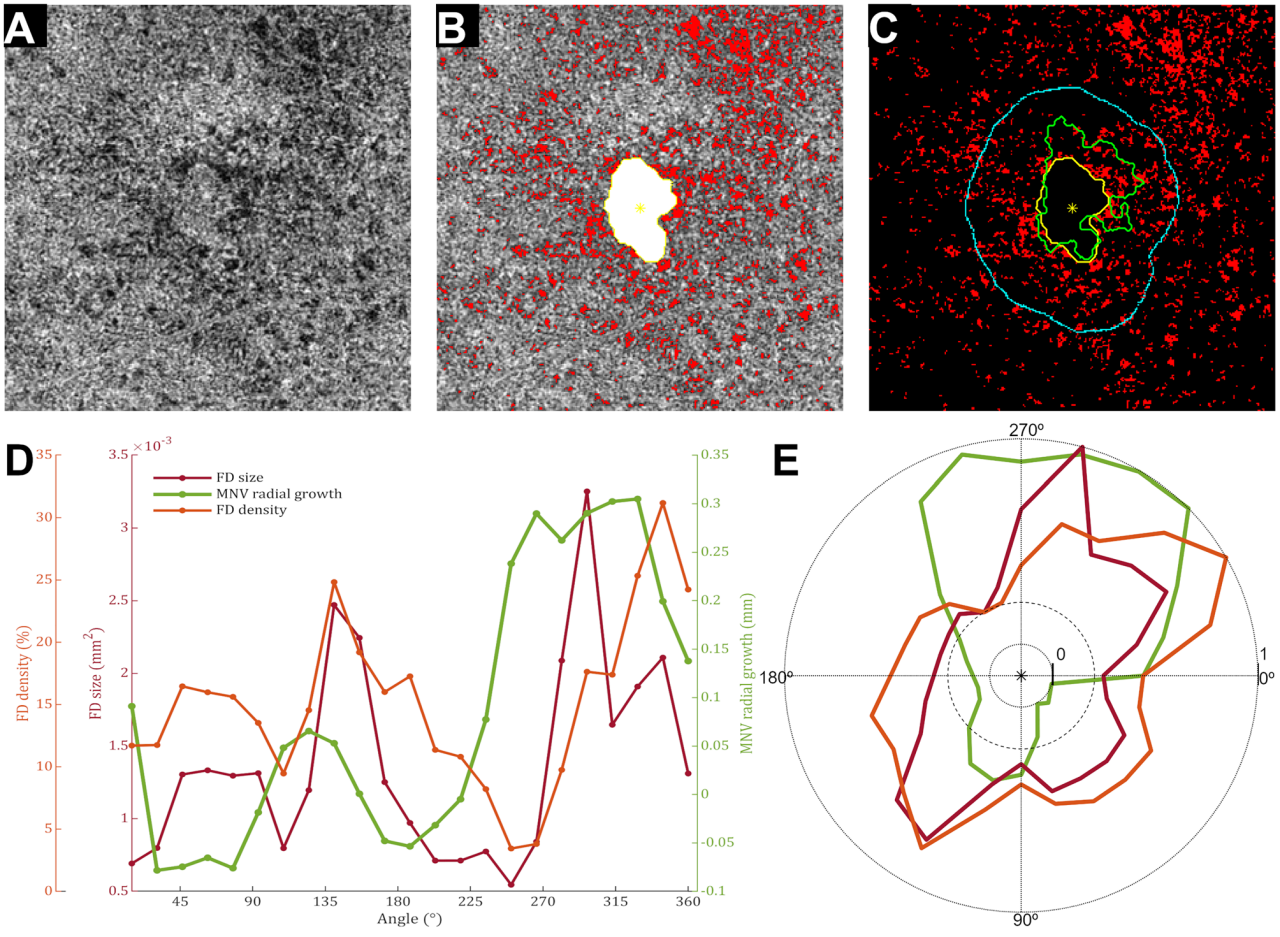
Single case analysis shows monotonic association between choriocapillaris (CC) flow deficits (FDs) features and neovascular growth patterns in half of the region of interest. (**A**) *En face* choriocapillaris (CC) image was obtained swept-source optical coherence tomography angiography. (**B**) False-colored CC flow deficits (FDs, red) were overlapped with macular neovascularization (MNV) segmentation (white), and its respective centroid (yellow asterisk) in the first visit. (**C**) Overlapping CC FDs (red) with MVN segmentation over follow-up (first visit, yellow; second visit, green) shows sectorial growth. (**D**) The plot of FD features (FD average size and FD percentage) and MNV patterns show a monotonic tendency between neovascular growth patterns and FD features within part of the image (180º–360º). Statistical analysis disclosed a weak correlation between MNV growth and FD average size (τ = 0.15, p = 0.35) and a very weak correlation between MNV growth and FD percentage (τ = 0.03, p = 0.88).

The single case analysis showed a significant correlation between FD features and neovascular growth in 70% of the cases and heterogeneous values of τ correlation coefficients that ranged from weak to strong correlation values. Figures 2 and 3 show typical cases with a significant correlation between neovascular growth and FD features. Although the analysis in 8 visits did not reach a significant correlation, an inspection of neovascular growth patterns and FD features disclosed a positive monotonic association in 180º out of 360º surrounding the neovascular network (Figs. 3 and 4). After averaging, the whole cohort analysis showed a weak positive correlation ($\overline{\tau }$ = 0.17, 95% CI [− 0.20, 0.62]) and a moderate positive correlation ($\overline{\tau }$ = 0.21, 95% CI [− 0.16, 0.68]) for the association between FD average size and FD % with neovascular growth, respectively.

## Discussion

In our analysis of 22 type 1 MNV identified in 19 patients with nvAMD, we observed a monotonic trend between neovascularization growth and CC FD average size and %. If a particular region showed increased CC FD size or %, it was likely to observe neovascular growth in that direction over follow-up. This trend was observed even in cases in which the correlation between neovascular growth and FD features did not reach statistical significance. These results suggest that there may be a trend between type 1 MNV growth directionality and nearby CC flow impairment. This pattern was observed irrespective of the location of the neovascularization (sub-foveal vs parafoveal). If the location relative to the foveal center was determinant, one would expect growth directionality to be different between sub-foveal and parafoveal type 1 MNV, but this was not observed. While we cannot discount the possibility that our findings are associated with other explanatory variables, we believe our results suggest that CC flow alterations influence the pattern of type 1 MNV growth in nvAMD.

Our findings are consistent with those of prior investigations suggesting that type 1 MNV may recapitulate areas of CC flow impairment^22^. We observed that, after a mean follow-up of 28 ± 3 months, 86% of the eyes showed type 1 MNV under the central fovea and relative preservation of the visual function (median BCVA: 0.25; interquartile range, 0.2–0.4 logMAR). All the patients received treatment during the follow-up, albeit with different treatment intervals. The herein reported functionality agrees with the data reported from several large datasets and supports that type 1 MNV might be able to support RPE and photoreceptor cells^5,9,23^.

Our findings might have some implications regarding the pathophysiologic processes of nvAMD. Some authors have noted that CC FDs’ average size in the central macula increases with aging, and is augmented in the fellow eyes of patients with nvAMD and in eyes that progress to MNV and cRORA^12–15,17^. In eyes with nvAMD, Moult et al. have shown non-uniformity of CC flow impairment surrounding type 1 MNV and suggested that angle-dependent and lesion-centered analyses would be appropriate for longitudinal studies^16^. Recently, Corvi et al. observed that in eyes with intermediate AMD that progressed to type 1 or 2 MNV there was focal impairment of the CC, contrary to eyes progressing to type 3 MNV or cRORA, in which the impairment was diffuse^14^. While our study corroborates those observations, we add additional information that may be useful for a better understanding of the association between neovascular growth and CC blood flow impairment.

We observed that CC FD features (size and %) were not uniform in the 600 µm ring area surrounding type 1 MNV, and we verified a trend of MNV growth over areas with increased average size and % of FD. These findings suggest there might be a perfusion-dependent expression of angiogenic factors driving neovascularization growth towards areas with larger FDs. While this neovascular growth pattern reached statistical significance in 70% of the cases, in the remainder, an association could only be perceived in half of the region surrounding the MNV and lowered the averaged correlation coefficients representing the entire cohort. We acknowledge that other factors potentially associated with growth directionality should be explored and might have accounted for these findings, including resistance in choroidal venous outflow and the type of deposits at the level of the RPE complex. We believe this topic should be further studied as it is likely to expand our understanding of the mechanisms involved in some MNV processes.

The main strengths of this study are long clinical follow-up and precision in the image analysis. To the best of our knowledge, this is the first study to assess the association between MNV growth patterns and CC flow analysis with a longitudinal design. The mean follow-up interval between the baseline and final SS-OCTA was 28 ± 3 months, which was greater than our predicted minimal timeframe (1 year) needed to observe significant changes in neovascular surface area growth on SS-OCTA^20^. Image analysis was based on a rigorous protocol that included semi-automatic segmentation of the retinal layers of interest in each B-scan and automated algorithms previously validated to perform registration of subsequent SS-OCTA acquisitions and to segment FDs and neovascular blood flow^10,24,25^.

This study was limited by the small sample size and limitations inherent to its retrospective design, which did not enable a uniform follow-up in all cases nor control diurnal variation or arterial pressure effects on the choriocapillaris. Although most patients had yearly SS-OCTA meeting inclusion criteria, in 9 cases (38%) the interval between SS-OCTA ranged between 2 and 3 years. Analyzing yearly SS-OCTA in these cases could have impacted the strength of the association between neovascular growth and FD quantitative measurements, albeit it is unlikely that it would have changed the monotonic association hereby reported. We also did not distinguish between different AMD subgroups (exudative vs non-exudative; treatment naive vs receiving treatment) given the low number of eyes per putative subgroup. Selecting cases with neovascular growth over follow-up also impacts the generalization of our results. We believe that evaluating FD features after type 1 MNV reaches a stable size can inform on the impact of choriocapillaris changes in neovascularization growth in future studies. We also acknowledge that the mean age of our cohort was 76 ± 2 years and 84% of the patients had systemic hypertension well controlled with medical treatment. As aging and systemic hypertension have been associated with an increased size of FD, we cannot exclude a contributory role. Additionally, the current study used non-averaged acquisitions performed by an SS-OCTA system. Although SS-OCTA is the more reliable technology for in vivo visualization of the CC, limitations inherent to its lateral resolution, sampling rate, and speckle noises must be acknowledged^10^. One strategy to mitigate the influence of speckle noises in CC quantification is using multiple-scan averaging. However, due to the study design, we used single scans for CC analysis, which is associated with an estimated mean bias of 4.5%^10^.

In conclusion, we observed a positive trend between the growth patterns of type 1 MNV and two surrogate markers of CC blood flow impairment, i.e., increased FD average size and FD %. Recapitulation of CC areas with blood flow impairment was associated with preserved visual function, which supports that type 1 MNV might be a compensatory process for restoring nutritional support to the macula, eventually becoming a highly effective AMD treatment capable of preserving central vision.

## Methods

A retrospective analysis of a consecutive case series of patients with neovascular AMD seen at regular intervals for routine monitoring and/or treatment with intravitreal anti-VEGF therapy by one retinal specialist (K.B.F) was performed at Vitreous Retina Macula Consultants of New York (New York, USA) between July and December 2021. This study was approved by the Western Institutional Review Board (Olympia, WA), written informed consent was obtained from each participant, and all methods were performed in accordance with the declaration of Helsinki.

Inclusion criteria consisted of patients with nvAMD managed on a treat and extend (TER)^26^ dosing regimen of anti-VEGF therapy in whom growth of type 1 MNV was documented with SS-OCTA with at least one 1-year interval. 1-year intervals were predicted to be sufficient to observe the evolution of treated type 1 MNV^20^. Baseline was defined as the first visit during which SS-OCTA identified type 1 MNV flow signal with a greatest linear dimension of ≥ 250 µm or 0.2 mm due to the difficulty of measuring these lesions reproducibly^21^. Since TER injection intervals ranged from 4 to 10 weeks, the visits closest to 12 months since the baseline or prior 1-year time point were included in the analyses. When 2 eyes of the same patient met eligibility, data from both eyes were included. Type 1 MNV was defined according to the Consensus Nomenclature for Reporting Neovascular Age-Related Macular Degeneration Data (CONAN) criteria^3^. Exclusion criteria included: previous treatment in the study eye with photodynamic therapy, high myopia (≥ − 6.0 diopters), a history of any other retinal disease deemed to affect SS-OCTA, complete retinal pigment epithelium and outer retinal atrophy (cRORA)^27^; a fibrovascular pigment epithelium detachment height > 250 µm; media opacities interfering with retinal imaging, eyes with any evidence of type 2 or 3 MNV, eyes with multiple type 1 MNV lesions, and eyes with neovascular lesions within 600 µm of the border of the SS-OCTA scan area.

All patients had undergone complete ophthalmologic examinations, including measurement of the best-corrected visual acuity (BCVA) using Snellen charts, slit-lamp biomicroscopy, indirect fundus ophthalmoscopy, structural SD-OCT (Spectralis HRA + OCT2 (Heidelberg Engineering, Heidelberg, Germany)) and SS-OCTA (PLEX Elite 9000 SS-OCT (Carl Zeiss Meditec, Inc, Dublin, CA)). The technical specifications of the SS-OCT are fully detailed elsewhere^21^. The treatment consisted of intravitreal injection of aflibercept (2.0 mg/0.05 ml) or ranibizumab (0.5 mg/0.05 ml). For this analysis, no distinction between either antiangiogenic drug was made.

For each eye, the SS-OCTA data analyzed was 6 × 6 mm (500 A-scans × 500 B-scans) SS-OCTA acquisitions centered on the fovea with a minimum signal strength index of 8, and minimal motion artifacts. SS-OCTA acquisitions from cases meeting inclusion criteria were exported as raw data (.IMG files) for image analysis. Prior to grading, SS-OCTA files were subjected to a preliminary review to identify artifacts (for example motion artifacts, signal shadowing due to intravitreal suspensions, etc.) affecting choriocapillaris imaging that would preclude reliable analysis. In cases considered potential candidates for an analysis of the choriocapillaris flow deficits, the segmentation of Bruch’s membrane was automatically performed by combined use of SS-OCT and SS-OCTA cubes and manually corrected if necessary, and the files were pre-processed to obtain an improved visualization and assessment of the choriocapillaris, as previously described^10^. Subsequently, SS-OCTA of subsequent visits was aligned with the baseline acquisition using an automated non-rigid registration algorithm, as previously described^10,24^.

SS-OCTA was processed to extract the neovascular contour outline and to segment choriocapillaris flow deficits. Image processing, image analysis, and statistical analysis were done using a code designed in MATLAB version R2020a (The MathWorks Inc., Natick, MA, USA). The neovascular contour was obtained using a previously described and validated automated algorithm that detected angiographic flow within the outer retina to the choriocapillaris (ORCC) slab after the removal of vessel projection artifacts^25^. An *en face* image of the choriocapillaris was obtained using a 16-µm thick slab with its anterior boundary located 4 µm beneath the Bruch’s membrane. Subsequently, flow deficits were segmented using a previously described and validated methodology^28,29^. The resulting neovascularization segmentation outlines were analyzed by two retina specialists. Cases in which there was disagreement on the MNV segmentation outline were excluded. A graphical representation of the different image processing steps is shown in supplemental Fig. 1.  Following previous conventions for the assessment of CC FD features in the vicinity of MNV^21^, a quantitative analysis of CC FD was conducted and correlated with neovascular growth over follow-up within a 600µm region extending from the neovascular contour in baseline scans. This analysis was performed using radial sectors centered on the MNV centroid, as previously postulated^16^. Two quantitative metrics were assessed: the CC FD percentage (%) and the average CC FD area. The CC FD% was defined as the percentage of pixels representing flow deficits relative to all the pixels within a sector. The average CC FD area within a sector was given in square millimeters (mm^2^). Neovascular growth was defined as the average difference in neovascularization outlines within a sector between visits spaced over more than 1 year and given in mm^2^. Neovascular growth and the corresponding values of FD features (% and average size) were computed for 23 sectors with 50% overlap, i.e. sectors measuring 30º (central angle) and overlapping half of adjacent sectors. Overlapping adjacent sectors enabled to mitigate the bias associated with iatrogenic FDs features, i.e., FDs spanned by the intersectoral borders, which has been considered a limitation of radial sectors analysis^16^. A graphical representation of the image analysis protocol is depicted in supplemental Fig. 2. The distribution of the variables of interest in the entire cohort was assessed using data plots. Before data plotting, the presence of outliers was assessed and removed using the isoutlier function for MATLAB. Afterward, FD features data was binned in uniform classes following the square root choice^30^, and the average neovascular growth for each interval (mean± standard error of the mean) was plotted and visually inspected. The association between FD features and neovascular growth was assessed using Kendall’s rank correlation coefficient (τ) and the resulting values were interpreted as previously described (less than 0.10: very weak; 0.10 to 0.19: weak; 0.20 to 0.29: moderate; and 0.30 or above: strong correlation)^31^. A τ value was obtained for each FD feature (% and average size) for each case. A global τ average for the entire cohort was calculated using Fisher’s Z transformation. Finally, an exploratory analysis of the demographic, clinical, and SS-OCTA (lesion size) data was conducted. Continuous variables are presented as mean ± standard deviation (SD) or median ± interquartile range (IQR) (Quartile 1 (Q1) and Quartile 3 (Q3)), as appropriate.

## Supplementary Information

Supplementary Information 1.

Supplementary Information 2.

Supplementary Information 3.

## Data Availability

The datasets generated during and/or analyzed during the current study are available from the corresponding author on reasonable request.
