# Supplementary figures and images for: Quantitative assessment of choriocapillaris flow deficits and type 1 macular neovascularization growth in age-related macular degeneration
Source: Sci Rep. 2023 May 26;13:8572. doi: 10.1038/s41598-023-35080-0 (PMC10220043; doi:10.1038/s41598-023-35080-0)

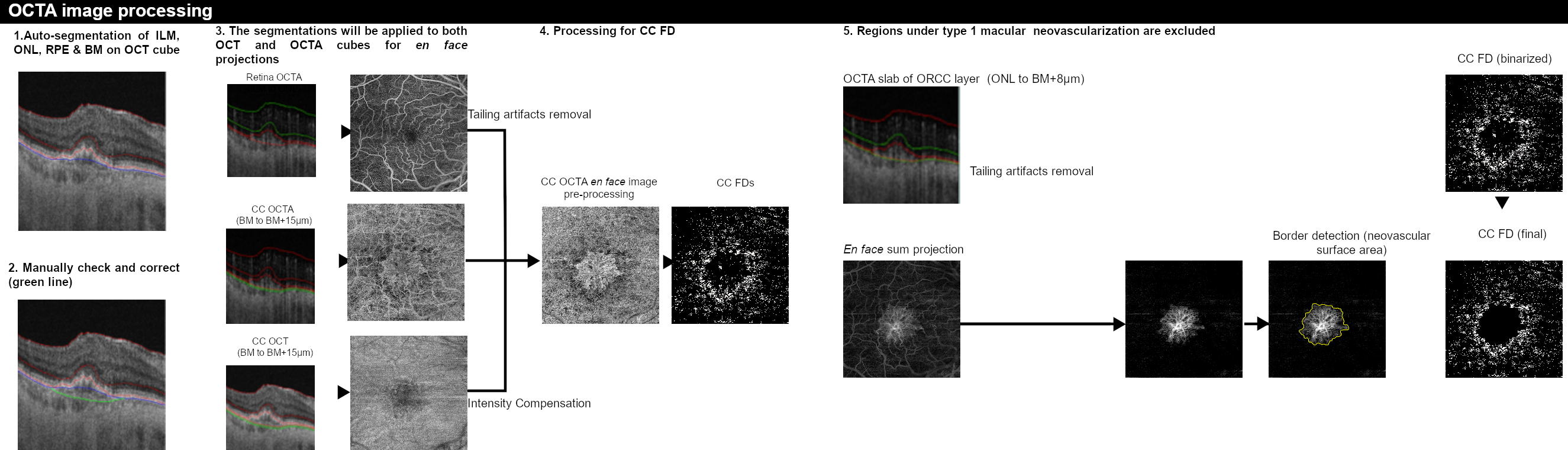

Supplement: Supplementary file 1 — Supplementary Information 1. [file 41598_2023_35080_MOESM1_ESM.tiff]
