# Supplementary material for: Quantitative assessment of choriocapillaris flow deficits and type 1 macular neovascularization growth in age-related macular degeneration
Source: Sci Rep. 2023 May 26;13:8572. doi: 10.1038/s41598-023-35080-0 (PMC10220043; doi:10.1038/s41598-023-35080-0)
Supplement: Supplementary file 3 — Supplementary Information 3. [file 41598_2023_35080_MOESM3_ESM.docx]

**Supplemental figure 1. Graphical representation of swept-source optical coherence tomography angiography (SS-OCTA) image processing steps.**

Following previous conventions for the assessment of CC FD features in the vicinity of MNV,^27^ a quantitative analysis of CC FD was conducted and correlated with neovascular growth over follow-up within a 600µm region extending from the neovascular contour in baseline scans. This analysis was performed using radial sectors centered on the MNV centroid, as previously postulated.^16^ Two quantitative metrics were assessed: the CC FD percentage (%) and the average CC FD area. The CC FD% was defined as the percentage of pixels representing flow deficits relative to all the pixels within a sector. The average CC FD area within a sector was given in square millimeters (mm^2^). Neovascular growth was defined as the average difference in neovascularization outlines within a sector between visits spaced over more than 1 year and given in mm^2^. Neovascular growth and the corresponding values of FD features (% and average size) were computed for 23 sectors with 50% overlap, i.e. sectors measuring 30º (central angle) and overlapping half of adjacent sectors. Overlapping adjacent sectors enabled to mitigate the bias associated with iatrogenic FDs features, i.e., FDs spanned by the intersectoral borders, which has been considered a limitation of radial sectors analysis.^16^ A graphical representation of the image analysis protocol is depicted in supplemental figure 2.

**Supplemental figure 2. Schematic representation of image processing and analysis**.

The distribution of the variables of interest in the entire cohort was assessed using data plots. Before data plotting, the presence of outliers was assessed and removed using the *isoutlier* function for MATLAB. Afterward, FD features data was binned in uniform classes following the square root choice^31^, and the average neovascular growth for each interval (mean± standard error of the mean) was plotted and visually inspected. The association between FD features and neovascular growth was assessed using Kendall’s rank correlation coefficient ($\tau$) and the resulting values were interpreted as previously described (less than 0.10: very weak; 0.10 to 0.19: weak; 0.20 to 0.29: moderate; and 0.30 or above: strong correlation).^32^ A $\tau$ value was obtained for each FD feature (% and average size) for each case. A global $\tau$ average for the entire cohort was calculated using Fisher’s Z transformation. Finally, an exploratory analysis of the demographic, clinical, and SS-OCTA (lesion size) data was conducted. Continuous variables are presented as mean ± standard deviation (SD) or median ± interquartile range (IQR) (Quartile 1 (Q1) and Quartile 3 (Q3)), as appropriate.
